# Undifferentiated pleomorphic sarcoma as a rare extracolonic manifestation in Lynch syndrome with MLH1 germline mutation: a case report

**DOI:** 10.3389/fonc.2026.1793258

**Published:** 2026-04-21

**Authors:** Yuxing Chen, Yilin Xiao, Ruihan Li, Mengjiao Wu, Yan Zong, Min Jin, Xiaona Chang, Hongli Liu

**Affiliations:** 1 Cancer Center, Union Hospital, Tongji Medical College, Huazhong University of Science and Technology, Wuhan, China; 2 Institute of Radiation Oncology, Union Hospital, Tongji Medical College, Huazhong University of Science and Technology, Wuhan, China; 3 Hubei Key Laboratory of Precision Radiation Oncology, Wuhan, China; 4 Department of Pathology, Union Hospital, Tongji Medical College, Huazhong University of Science and Technology, Wuhan, China

**Keywords:** Lynch syndrome, mismatch repair deficiency, MLH1 variant, synchronous malignancies, undifferentiated pleomorphic sarcoma

## Abstract

Undifferentiated pleomorphic sarcoma (UPS) is a rare and highly aggressive soft tissue sarcoma that typically occurs in the extremities but is rarely found in the retroperitoneal cavity. Despite the documented cases of sarcomas in patients with Lynch syndrome (LS), the relationship between these malignancies and LS has not yet been fully elucidated. In this report, we present the case of a patient with LS who harbored a germline MLH1 loss-of-function mutation (c.2171T>A) and developed synchronous malignancies, including colorectal cancer, cholangiocarcinoma, and retroperitoneal UPS. Consequently, molecular testing revealed mismatch repair deficiency (dMMR) among two cancers, thereby confirming the diagnosis of LS. This case suggests that UPS may represent a rare extracolonic manifestation of LS and highlights the importance of considering MMR/MSI testing in sarcomas of uncertain origin, which may have implications for diagnosis and personalized treatment strategies, including the use of immune checkpoint inhibitors.

## 1 Introduction

Undifferentiated pleomorphic sarcoma (UPS) is a high-grade soft tissue sarcoma that most frequently occurs in the extremities of older adults and is characterized by marked cellular pleomorphism and the absence of definitive lineage differentiation. UPS represents a rare form of malignancy and accounts for approximately 5–10% of all soft tissue sarcomas (1). Recent studies estimate that the annual incidence of UPS in the United States ranges from 0.06 to 0.67 cases per 100,000 individuals (2). Retroperitoneal UPS is exceptionally rare and poses unique diagnostic and therapeutic challenges because of its anatomical complexity and aggressive behavior.

Lynch syndrome (LS), also known as hereditary nonpolyposis colorectal cancer (HNPCC), is a common hereditary cancer syndrome that is primarily associated with colorectal cancer (CRC) and other malignancies (3, 4). As a familial genetic disorder characterized by mismatch repair (MMR) protein deficiency, LS is linked to mutations in four key MMR genes: MLH1, MSH2, MSH6, and PMS2 (5). These genetic alterations significantly increase the risk of cancer development in affected individuals. The spectrum of LS can be categorized into narrow and broad. The narrow spectrum primarily includes CRC and endometrial cancer, while the broad spectrum encompasses gastric cancer, ovarian cancer, nervous system tumors, and cutaneous malignancies (6). Clinically, 92% of male and 74% of female patients with LS present with CRC (3). Special attention should be given to patients with a personal or family history of CRC, as they may have an underlying LS diagnosis. At present, two main diagnostic approaches are used for LS. The first such approach involves immunohistochemistry (IHC) to detect MMR proteins and microsatellite instability (MSI) testing to identify MMR protein loss or microsatellite instability-high (MSI-H) status. The second approach consists of direct germline genetic testing for individuals with a personal or family history suggestive of LS. Defects in these genes lead to the failure of DNA mismatch repair, which results in increased genomic instability and the promotion of tumorigenesis (7).

Although sarcomas are not traditionally included within the LS tumor spectrum, accumulating evidence suggests that mismatch repair deficiency (dMMR) may contribute to the pathogenesis of certain sarcoma subtypes (8). Recent studies and systematic reviews have reported that the majority of sarcomas in LS patients exhibit dMMR or MSI-H status, and of the affected genes, MSH2 is the most frequently altered gene (9, 10). These findings expand the clinical spectrum of LS beyond that of classic colorectal and endometrial carcinomas to include rare extracolonic manifestations such as soft tissue sarcomas. However, documented cases of UPS arising in the context of LS remain exceedingly scarce, and comprehensive molecular characterization is limited. Prior studies have described a handful of LS-associated sarcomas—predominantly leiomyosarcoma and malignant fibrous histiocytoma—with variable dMMR and uncertain causal relationships. To our knowledge, no previous report has described an extracolonic UPS with a confirmed pathogenic MLH1 germline mutation.

Here, we report a molecularly confirmed case of LS in a 60-year-old male who harbored a germline MLH1 c.2171T>A (p.L724*) variant and who developed synchronous colorectal cancer, cholangiocarcinoma, and retroperitoneal UPS. This case provides novel evidence that indicates UPS as a potential extracolonic manifestation of LS and highlights the clinical importance of comprehensive molecular testing—including MSI, and germline variant analysis—in the diagnostic evaluation of atypical LS-associated tumors.

## 2 Patient description

**Figure 1:**
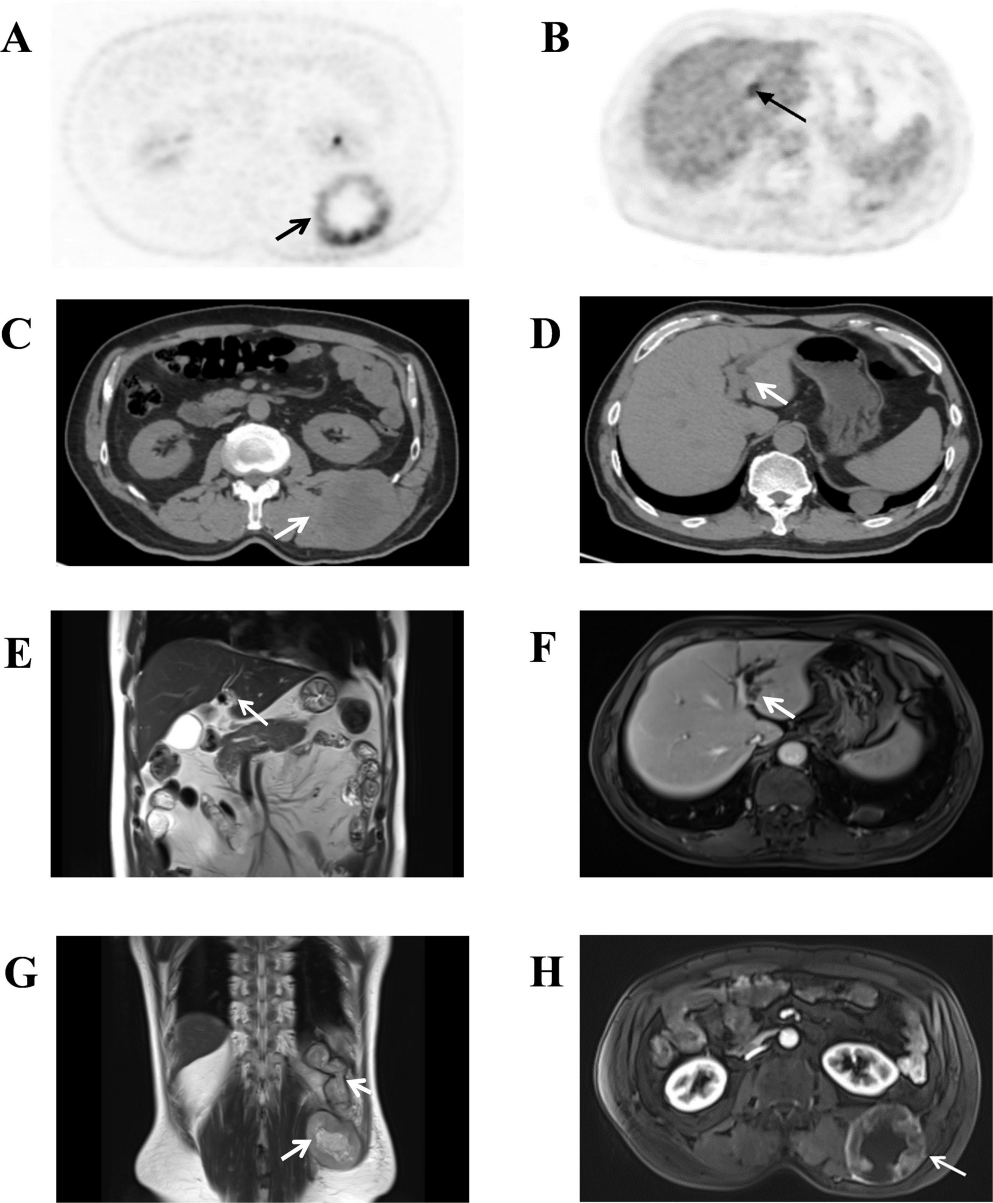
Pertinent imaging studies of the patient. **(A-D)** PET-CT demonstrated hypermetabolic masses in the left dorsal subcutaneous tissue(SUVmax11.0) **(A, C)** and the left hepatic lobe(SUVmax4.1) **(B, D-H)** Coronal T2-weighted imaging (T2WI) and axial arterial phase contrast-enhanced T1-weighted imaging (T1WI) magnetic resonance imaging (MRI) of the liver **(E, F)** and retroperitoneal masses **(G, H)**. The longest dimension of the hepatic tumor was approximately 1.5 cm, it exhibits a slightly prolonged T2WI signal and shows relative hypointense enhancement compared to normal liver parenchyma. The vertical dimension of the sarcoma was approximately 13.2 cm, with a maximum cross-sectional size of approximately 7.0 × 6.4 cm; T2WI shows mixed signal intensity. On T1-weighted contrast-enhanced scans, there is a central area of non-enhancing necrosis, and the peripheral solid component demonstrates heterogeneous enhancement.

In April 2024, a 60-year-old male patient developed an egg-sized, painless mass on his back three days after receiving a local massage. Ultrasonography at a community clinic suggested a hematoma, and no treatment was initiated. However, the lesion continued to increase in size and became painful over the next three months. Magnetic resonance imaging (MRI) subsequently revealed a malignant neoplasm. Further positron emission tomography–computed tomography (PET–CT) revealed a left retroperitoneal mass (central hypodensity, heterogeneous metabolic activity; SUVmax 11.0) 70×64 mm in size, with mild focal metabolic activity inferior to the left latissimus dorsi (SUVmax 2.4). A hypodense left hepatic lesion 15×10 mm in size demonstrated increased metabolism (SUVmax 4.1) (**Figures 1A–D**). MRI confirmed tumor involvement of the erector spinae and latissimus dorsi, with consideration given to the possibility of a hepatic tumor (**Figures 1E–H**). In the absence of any metastatic lesions at this time, the patient underwent extensive en bloc tumor resection with negative surgical margins (R0 resection). No adjuvant radiotherapy was administered postoperatively. Notably, the patient underwent radical colectomy for colonic adenocarcinoma and received 6 cycles of adjuvant chemotherapy in December 2000. In August 2008, he underwent radical resection for rectal adenocarcinoma, followed by 5 cycles of adjuvant chemotherapy with the FOLFOX regimen. The patient’s father, paternal aunt, and two cousins were diagnosed with colorectal cancer and underwent surgical treatment; however, none underwent genetic testing. The patient’s father, paternal aunt, and elder cousin died of rectal cancer, whereas the younger cousin remains alive with no recurrence or metastasis after surgery.

## 3 Diagnosis

Based on the imaging findings, a malignant retroperitoneal tumor was strongly suspected. The large mass located in the left retroperitoneal region demonstrated heterogeneous metabolic activity on PET-CT (SUVmax 11.0) and showed infiltrative growth involving the erector spinae and latissimus dorsi muscles on MRI. These radiological characteristics suggest the presence of an aggressive high-grade soft-tissue sarcoma rather than a benign soft-tissue lesion. For the differential diagnosis of this retroperitoneal mass, several primary retroperitoneal sarcomas, such as liposarcoma, leiomyosarcoma and malignant peripheral nerve sheath tumors, need to be considered. Given the infiltrative margins, heterogeneous internal signal intensity, and areas of necrosis, a high-grade undifferentiated sarcoma was considered the most likely diagnosis.

In addition to the retroperitoneal tumor, PET-CT revealed a metabolically active lesion in the left hepatic lobe (SUVmax 4.1), raising suspicion of either metastatic disease or a second primary hepatic malignancy. Because the liver lesion was single and small at that time, we initially determined that the liver lesion was not related to the retroperitoneal mass. The hepatic lesion was independent of the retroperitoneal mass.

The above imaging characteristics directly informed surgical planning by highlighting the need for wide en bloc resection and careful assessment of deep margins. The tumor was subsequently completely removed, and postoperative histopathological examination confirmed the presence of two distinct primary tumors: a retroperitoneal undifferentiated pleomorphic sarcoma and an intrahepatic cholangiocarcinoma. These findings established the diagnosis of synchronous primary malignancies. Moreover, considering the patient’s multiple primary colorectal cancers and strong family history of colorectal malignancy, LS was highly suspected.

## 4 Gross and pathological features

**Figure 2:**
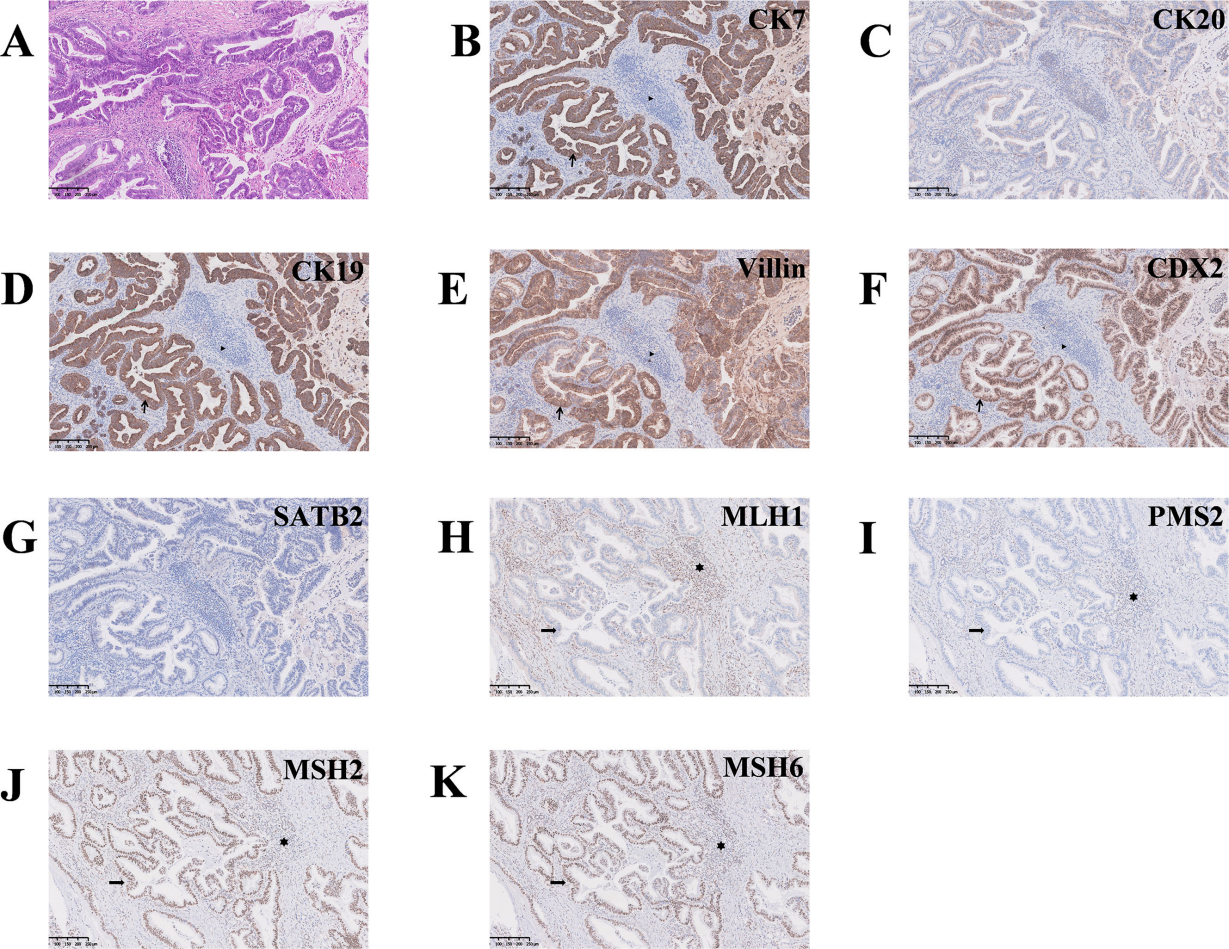
Histopathological characteristics and immunohistochemical profile of the hepatic mass. **(A)** The hepatic tissue revealed an irregular glandular architecture and atypical tumor cells, magnification ×100. **(B-G)** Hepatic tumor cells were strongly positive for CK7 (**(B)** magnification ×100), CK19 [**(D)** magnification ×100], Villin [**(E)** magnification ×100], and CDX2 [**(F)** magnification ×100], with focal positivity for CK20 [**(C)** magnification ×100] and SATB2 [**(G)** magnification ×100], supporting a gastrointestinal or pancreatobiliary origin; clinicopathological correlation was required to exclude metastatic disease. **(H-K)** Loss of MLH1 [**(H)** magnification ×100] and PMS2 [**(I)** magnification ×100] expression with retained MSH2 [**(J)** magnification ×100] and MSH6 [**(K)** magnification ×100] expression indicated dMMR. In the appropriate clinical context, these findings support the diagnosis of primary cholangiocarcinoma with dMMR and raise suspicion for LS.

**Figure 3:**
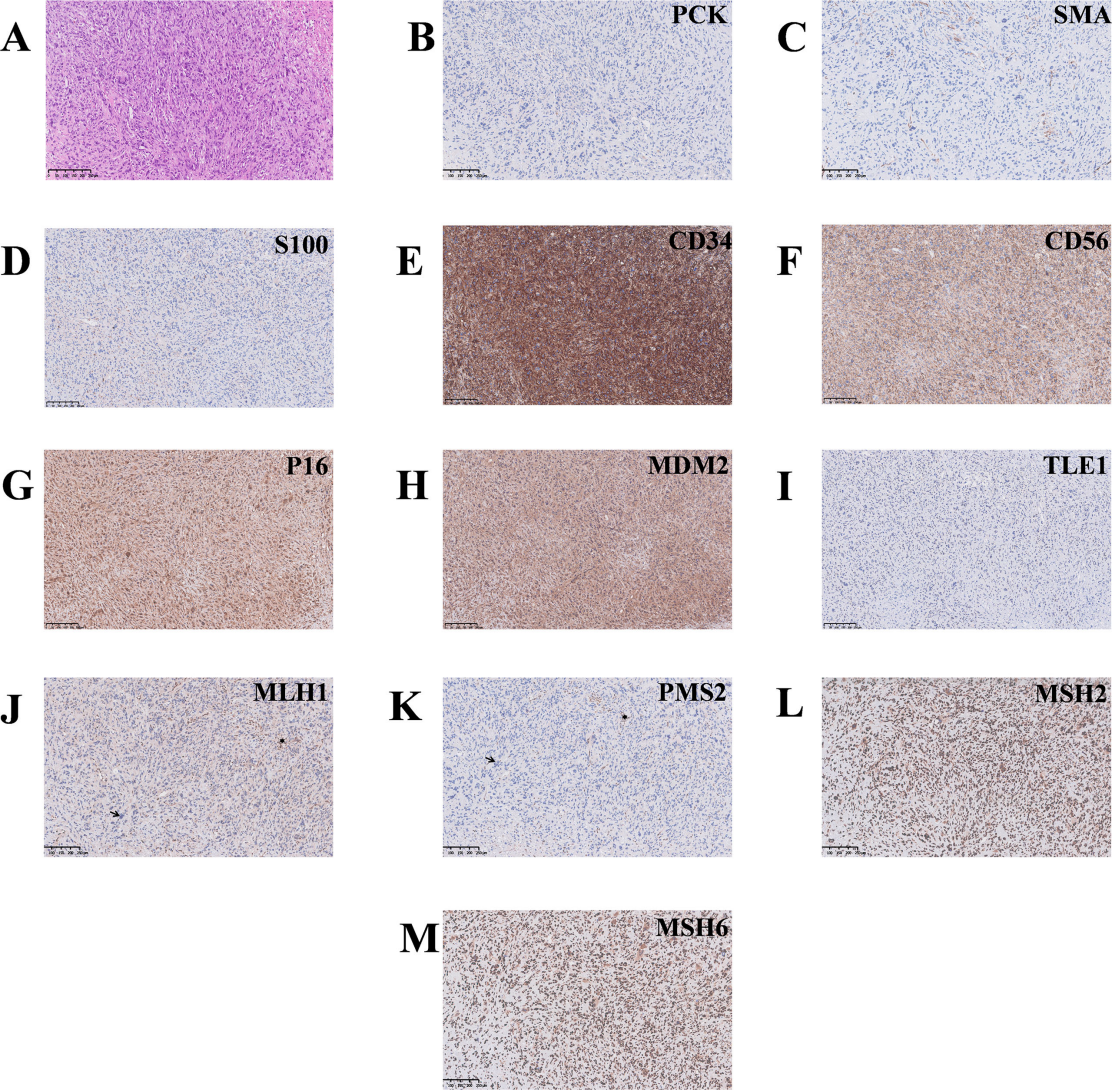
Histopathological characteristics and immunohistochemical profile of the retroperitoneal mass. **(A)** The retroperitoneal tissue demonstrated bundles of spindle cells with focal disorganization and exhibited elongated or fusiform nuclei and scant stromal components; magnification ×100. **(B-I)** IHC demonstrated that the tumor cells were negative for epithelial markers (PCK, **(B)** magnification ×100), smooth muscle markers (SMA, **(C)** magnification ×100), neural markers S100 (**(D)** magnification ×100), and MDM2 (**(H)** magnification ×100), excluding carcinoma, leiomyosarcoma, malignant peripheral nerve sheath tumor, and dedifferentiated liposarcoma. The tumor cells showed positivity for CD34 (**(E)** magnification ×100), CD56 (**(F)** magnification ×100), and P16(**(G)** magnification ×100), with focal weak staining for TLE1(**(I)** magnification ×100); however, these findings were not lineage-specific. **(J-M)** Loss of MLH1 (**(J)**, magnification ×100) and PMS2 (**(K)**, magnification ×100) expression with retained MSH2 (**(L)**, magnification ×100) and MSH6 (**(M)**, magnification ×100) expression indicated dMMR. Taken together, these findings support the diagnosis of high-grade UPS and suggest a possible association with LS.

The retroperitoneal tumor measured 11.5 cm × 7.5 cm in size. Gross examination revealed a well-demarcated, homogeneous, moderately firm cut surface, ranging from gray–yellow to gray–white in color, with areas containing bone tissue. The hepatic tissue contained a 2.0 cm × 1.7 cm gray–yellow nodule adjacent to the resection margin. Histopathologic examination of both specimens revealed distinct morphologic features: The liver tissue sections demonstrated numerous irregularities and variably sized glands arranged in a disorganized pattern, and neoplastic cells exhibited marked atypia, prominent mucinous pools, and stromal desmoplasia, which is consistent with moderately differentiated adenocarcinoma with mucinous lakes (**Figure 2A**). In contrast, the proliferation of densely packed spindle cells arranged in fascicular and storiform patterns (with haphazard areas) was detected in the retroperitoneal mass sample. The mass also exhibited cellular pleomorphism (variation in size and shape) and spindled to elongated (cigar-shaped) nuclei, including occasional tumor giant cells; its stromal component was relatively scant and was composed primarily of interlacing fibrous tissue interspersed among the spindle cells (**Figure 3A**).

Immunohistochemical staining with appropriate positive and negative controls was performed on both the retroperitoneal mass and liver samples. The immunohistochemical markers applied in this case are primarily intended for the differential diagnosis of sarcomas. In routine clinical practice, these markers are not quantitatively assessed for percentage positivity or staining intensity; therefore, detailed semiquantitative IHC scores are not typically generated and are unavailable for this case. With respect to the retroperitoneal tumor, the neoplastic cells were negative for PCK, CK8/18, SOX10, S100, SMA, Desmin, MyoD1, Myogenin, and MDM2 but were positive for CD34, CD56 and P16, with weak focal positivity for TLE1 (**Figures 3B–I**).

Based on histopathological and immunohistochemical findings, the retroperitoneal tumor was diagnosed as UPS. Histologically, the tumor exhibited a high-grade spindle cell morphology with marked nuclear pleomorphism and frequent atypical mitotic figures. The proliferative index, as assessed by Ki-67, was markedly elevated (~70%), and P53 showed a mutant-type expression pattern, indicating high proliferative activity and aggressive biological behavior. Immunohistochemical analysis demonstrated that the tumor cells lacked expression of lineage-specific differentiation markers. Epithelial markers (PCK, CK8/18) and p40 were negative, excluding carcinoma and sarcomatoid carcinoma. Smooth muscle markers (desmin and SMA) were not expressed, arguing against leiomyosarcoma. Neural crest–related markers (S100 and SOX10) were also negative, excluding malignant peripheral nerve sheath tumor and other neurogenic tumors. In addition, only focal weak positivity for TLE1 and negative ALK expression did not support synovial sarcoma or inflammatory myofibroblastic tumor. Dedifferentiated liposarcoma was effectively excluded based on the absence of MDM2 expression in conjunction with the lack of morphological evidence of lipogenic differentiation. Although CDK4, CD34, and p16 showed focal or partial positivity, these markers are not lineage-specific and can be observed in a variety of mesenchymal neoplasms; therefore, they were not considered diagnostically decisive. Similarly, CD56 positivity was regarded as nonspecific and did not indicate a particular line of differentiation. Taken together, in the absence of any definable line of differentiation and after excluding other specific sarcoma subtypes, the tumor was ultimately classified as a high-grade UPS.

With respect to the hepatic tumor, the neoplastic cells were positive for CK7, CK19, Villin, and CDX2 and exhibited focal positivity for CK20 and SATB2 (**Figures 2B–G**). This immunophenotype is not supportive of classical metastatic colorectal carcinoma to the liver. However, given the overlapping immunophenotype between cholangiocarcinoma and primary gastrointestinal tract carcinomas, correlation with clinical and imaging findings is needed to exclude a metastatic lesion from a gastrointestinal (or other) primary site before a diagnosis of primary cholangiocarcinoma is confirmed. Notably, the tumor cells in both specimens demonstrated an MMR protein deficiency pattern, with loss of MLH1 and PMS2 expression and sustained MSH2 and MSH6 expression (MLH1[-], PMS2[-], MSH2[+], MSH6[+]) (**Figures 3J–M**, **2H–K**). This MMR-deficient profile is suggestive of LS; however, a definitive diagnosis requires correlation with germline genetic testing results.

## 5 Imputability of Lynch syndrome

Next-generation sequencing (NGS) of the cholangiocarcinoma tissue and corresponding normal liver tissue using a large-panel PANL-1021 gene assay revealed a high tumor mutation burden (TMB) of 38.4 mutations per megabase (mut/Mb) and an MSI-H status. In the normal tissue sample, a heterozygous germline variant, MLH1c.2171T>A (p.L724*), a class IV (likely pathogenic) variant, was identified. This truncating variant is predicted to result in loss of protein function, which is consistent with the observed loss of MLH1 protein expression by immunohistochemistry (MLH1-). This variant was not reported in population germline variant databases, and while it was registered in the ClinVar database, computational predictive software did not provide an assessment for this specific locus. In addition, notable somatic variants, including KRAS (c.183A>T) and NTRK1 (c.1632 + 1G>A), were identified. The detailed NGS results are summarized in **Table 1**. Overall, the MSI-H status, loss of MLH1/PMS2 expression on immunohistochemistry, and identification of a germline MLH1 likely pathogenic variant collectively support the diagnosis of LS in this patient.

**Table 1:** Summary of NGS findings in the cholangiocarcinoma.

Category	Gene	Alteration	Variant type	Allele frequency/zygosity	Clinical significance
Somatic mutation	KRAS	c.183A>T (p.Q61H)	Missense (Exon 3)	0.7%	Likely oncogenic driver mutation (II)
Germline mutation	MLH1	c.2171T>A (p.L724*)	Nonsense (Exon 19)	Heterozygous	Likely pathogenic (IV)
Somatic mutation	NTRK1	c.1632 + 1G>A	Splice-site (Intron 13)	1.6%	Variant of uncertain significance (III)
Genomic feature	:	Microsatellite instability (MSI)	:	:	MSI-H
Genomic feature	:	Tumor mutational burden (TMB)	:	38.4 muts/Mb	High

## 6 Results

**Figure 4:**
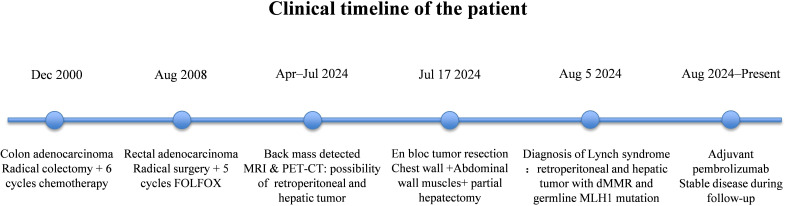
Clinical timeline of the patient from the first colorectal cancer diagnosis to the current treatment and follow-up.

Following a thorough review of the patient’s medical history and a comprehensive set of diagnostic tests, the following diagnoses were established: retroperitoneal UPS (T2N0M0), characterized by dMMR based on loss of MLH1/PMS2 expression, and LS with a MLH1 variant (c.2171T>A). On July 17, 2024, the patient underwent surgical resection, which included removal of the chest wall lesion, partial rib resection, diaphragmatic resection and reconstruction, partial erector spinae resection, left hemihepatectomy, and cholecystectomy. One month after surgery, the patient underwent follow-up MRI of the hepatobiliary system, retroperitoneum, and pelvis at our hospital and achieved good postoperative recovery. After contraindications to immunotherapy were considered, the patient started pembrolizumab (200 mg every three weeks) therapy on August 17, 2024, and has continued treatment to date. A detailed timeline of the patient’s clinical course is shown in **Figure 4**. At the most recent follow-up (approximately 19 months after treatment initiation), the patient continues to receive therapy without evidence of disease recurrence.

## 7 Discussion

UPS is a highly heterogeneous malignant tumor that is characterized by pleomorphic cellular morphology, a lack of definitive histological differentiation, and vascular proliferation (11, 12). It is most commonly observed in adults, particularly middle-aged and elderly individuals, and typically arises in the extremities and trunk (13). The histopathological features of UPS are nonspecific, and owing to its diverse cellular composition and absence of distinct tissue markers, the diagnosis is generally made by exclusion (1, 14). Key morphological characteristics of UPS include marked cellular heterogeneity, nuclear atypia, and an absence of clear tissue differentiation, which are often accompanied by necrosis, hemorrhage, and vascular proliferation. Tumor cells exhibit atypical pleomorphic spindle-shaped morphology with frequent mitotic figures. The invasive depth of tumors can extend deep into the dermis, subcutaneous tissue, fascia, and skeletal muscle (15). Within the fibrous stroma, the tumor may present in a storiform, fascicular, or sheet-like arrangement (14). On the basis of the depth of invasion, UPSs can be classified as either superficial or deep tumors, and the boundary is typically defined by the superficial fascia and other organ structures. Notably, deep-seated UPS is associated with poor prognosis (15).

In clinical practice, the diagnosis of UPS is made by exclusion, as this tumor must be differentiated from other soft tissue sarcomas, such as dedifferentiated liposarcoma, pleomorphic leiomyosarcoma, malignant peripheral nerve sheath tumor, and pleomorphic rhabdomyosarcoma. These tumors can share overlapping histological features, but key differences have been observed in their immunohistochemical profiles and molecular characteristics that aid in diagnosis.

Dedifferentiated liposarcoma is characterized by lipogenic areas and MDM2/CDK4 amplification, which are absent in UPS (16). In our case, MDM2 amplification testing was negative, which excluded this diagnosis. Pleomorphic leiomyosarcoma typically expresses SMA or Desmin, whereas UPS lacks smooth-muscle differentiation, and this tumor is negative for both markers. Malignant peripheral nerve sheath tumors may be positive for S100 or SOX10, which indicates the presence of Schwannian differentiation, but these markers are absent. Pleomorphic rhabdomyosarcomas express myogenic markers such as Desmin, MyoD1, and Myogenin, none of which are expressed in this case (17). Considering the lack of MDM2 amplification and the absence of smooth-muscle, neural, or myogenic differentiation, the findings supported a final diagnosis of UPS.

Sarcoma cases that originate as a result of LS have rarely been reported, and currently, sarcomas are not officially included in the LS tumor spectrum (18). As mesenchymal tumors have not traditionally been regarded as part of the LS tumor spectrum, their potential association with LS has not been fully recognized. Consequently, a significant proportion of sarcomas are not routinely evaluated for mismatch repair deficiency or microsatellite instability, which may contribute to the insufficient identification of LS-associated sarcomas. Angela et al. investigated two LS families in Germany and interviewed 340 individuals from 23 LS families with pathogenic germline variants and collected relevant information on their first- and second-degree relatives. Among these families, germline mutations in MLH1 and MSH2 were identified in 9 and 11 families, respectively. Notably, the recorded malignancies included two cases of malignant fibrous histiocytoma, one case of leiomyosarcoma, one case of gliosarcoma, and one case of osteosarcoma—tumors not traditionally associated with LS (3). Further MSI PCR testing revealed that two patients with LS and malignant fibrous histiocytoma harbored germline MSH2 mutations (MSH2 c.2038CCT or MSH2 c.942+-3ACT) within their respective families (3). These findings suggest that while sarcomas are not yet officially included in the LS tumor spectrum, individuals with LS may have an increased risk of developing sarcomas.

Among 95 reported cases of sarcomas in LS between 2000 and 2022, nearly half were documented within the past five years (19). UPS, leiomyosarcoma, and liposarcoma were the most frequently observed histological subtypes. Notably, 81% of sarcomas exhibited dMMR, and 77% demonstrated MSI-H, which is consistent with the molecular characteristics of LS-associated malignancies (19). In the diagnostic evaluation of sarcoma patients with a prior history of colorectal cancer, LS should not be overlooked. Assessment of the MMR and MSI status of sarcomas is essential for guiding both diagnostic and therapeutic strategies (18). When our patient was diagnosed with colorectal cancer twice in the early stages, because universal mismatch repair defect screening for colorectal cancer had not been widely implemented in the clinical environment in China at that time and because molecular diagnostic methods such as immunohistochemical detection of MMR proteins and MSI testing had just been incorporated into the standard diagnostic process, the patient did not undergo screening for LS. Consequently, despite the patient having early-onset colorectal cancer and a family history, the possibility of LS was not formally evaluated during the initial onset of the disease.

Surgical resection remains the primary treatment for LS-associated UPS (20, 21), whereas systemic therapy and immunotherapy have emerged as potential therapeutic approaches. Anthracycline-based chemotherapy is currently the standard systemic treatment for UPS; however, its efficacy in advanced-stage disease remains suboptimal (21, 22). Single-agent doxorubicin was compared with doxorubicin plus ifosfamide in a phase III randomized controlled trial. Compared with monotherapy, combination therapy resulted in a significantly higher objective response rate (ORR) and median progression-free survival (PFS) (26% vs. 14% and 7.4 months vs. 6.4 months, respectively). However, the median overall survival (OS) did not significantly improve (14.3 months vs. 12.8 months). Additionally, combination therapy was associated with greater drug toxicity and a higher incidence of grade 3 and 4 adverse events. Given the increased toxicity and the lack of substantial overall survival benefit, single-agent doxorubicin remains the preferred treatment for advanced UPS (23). Furthermore, tyrosine kinase inhibitors, antiangiogenic agents, and drugs targeting the cell cycle, tumor microenvironment, and signal transduction pathways have shown promising therapeutic potential in UPS treatment (22).

Apart from conventional chemotherapy, immune checkpoint inhibitors have been shown to have substantial efficacy in tumors with dMMR or MSI-H. An extension study based on KEYNOTE-016 evaluated the efficacy of PD-1 blockade therapy in 12 different tumor types (all MMR-deficient advanced cancers). An objective radiological response was observed in 53% of patients, whereas 21% demonstrated a complete response. The 1-year and 2-year PFS and OS were significantly greater than the expected values for the same cohort of advanced patients (24). In addition, based on pooling data from several clinical trials, including KEYNOTE-016 and KEYNOTE-164, the U.S. Food and Drug Administration approved pembrolizumab for the treatment of unresectable or metastatic MSI-H/dMMR solid tumors regardless of tumor origin in 2017 (25). In view of these findings, it is important to consider whether analogous immunotherapeutic strategies could be effective with respect to sarcomas with dMMR or MSI-H. Unlike other sarcomas, UPS is characterized by greater heterogeneity, greater immune infiltration, and relatively increased PD-1 expression (21, 26). In LS patients, the loss of MMR-related genes leads to dMMR and MSI-H, resulting in the accumulation of genetic mutations and the generation of neoantigens (27, 28), enhancing tumor recognition by the immune system. Several clinical trials have demonstrated promising clinical activity of immune checkpoint inhibitors in soft tissue sarcomas. The SARC028 phase II trial evaluating pembrolizumab in advanced soft tissue sarcoma reported objective responses, particularly in UPS and dedifferentiated liposarcoma (29). Similarly, the Alliance A091401 study demonstrated clinical activity of nivolumab alone or in combination with ipilimumab in metastatic sarcoma (30). Notably, the majority of these studies evaluated immune checkpoint inhibitors in patients with advanced or metastatic disease rather than in the adjuvant setting. In the context of LS, additional evidence also supports the potential role of immunotherapy in sarcomas. A nationwide retrospective study in France included 81 LS patients with sarcomas, of whom 36% were diagnosed with UPS. Among patients who underwent immunohistochemical and/or molecular testing, 75% exhibited dMMR, and 45% demonstrated MSI-H. Eight patients received treatment with pembrolizumab or nivolumab, achieving an ORR of 50%, and of these, three patients experienced a complete radiological response. One patient achieved a pathological complete response, and the duration of treatment response ranged from 6 to 20 months (31). Similarly, a report from the UCLA Comprehensive Cancer Center described three LS patients with dMMR sarcomas who received PD-1 inhibitors after surgery. In this context, two patients did not experience disease progression, whereas one patient with advanced UPS experienced disease progression after 10 months (32). These outcomes exceeded the ORR and progression-free survival observed in MMR-proficient patients treated with immunotherapy. Overall, immunotherapy, particularly pembrolizumab, has demonstrated significant clinical efficacy in LS-associated soft tissue sarcomas. In dMMR/MSI-H sarcomas, immunotherapy is associated with a high ORR and a prolonged duration of response. Although evidence supporting the use of immune checkpoint inhibitors in the adjuvant setting for sarcoma remains limited, the presence of dMMR/MSI-H status in our patient suggested potential sensitivity to PD-1 blockade. Therefore, pembrolizumab was selected as a systemic adjuvant therapy following complete surgical resection to reduce the risk of recurrence. This therapeutic rationale was primarily based on the dMMR/MSI-H status in this patient, whereas no TMB data were available for the UPS component.

In this case, MSI status was determined using a large-panel NGS assay rather than conventional PCR-based testing. Although PCR-based assays have historically been considered the gold standard, several studies have demonstrated that NGS-based approaches can reliably detect MSI across multiple tumor types (33–35). In our patient, the MSI-H result was further supported by the presence of a pathogenic MLH1 germline mutation and loss of MLH1/PMS2 expression on immunohistochemistry, confirming a deficient mismatch repair phenotype. At treatment initiation, the patient exhibited no residual or recurrent disease on MRI following complete surgical resection. After contraindications to immunotherapy were considered, pembrolizumab (200 mg every three weeks) was initiated on August 17, 2024, as an adjuvant therapy to eradicate potential micrometastatic disease and reduce the recurrence risk. Disease evaluation was performed using the RECIST 1.1 criteria at 12-week intervals. Clinical monitoring, physical examination, and laboratory testing were conducted before each infusion to assess immune-related adverse events (irAEs), including dermatologic, gastrointestinal, endocrine, and pulmonary toxicity. The patient tolerated pembrolizumab well, with only grade 1 fatigue and no significant irAEs noted. After six months of treatment, the patient did not experience disease progression and had stable imaging findings and preserved performance status.

The long-term outcomes of patients with dMMR/MSI-H sarcomas treated with immunotherapy suggest a sustained benefit but also highlight the risk of acquired resistance. To ensure early detection of recurrence or immune-related toxicity, a structured surveillance plan was implemented, consisting of MRI or PET-CT every three months for the first two years and every six months thereafter, combined with routine hematologic, hepatic, renal, and thyroid function testing. Molecular reassessment of MMR protein expression, and PD-L1 status may be considered during progression to inform subsequent immunotherapy or combination approaches.

Overall, this report broadens the range of tumors associated with LS by identifying UPS as a rare extracolonic manifestation linked to a germline MLH1 mutation. It also highlights the diagnostic value of routine MMR/MSI testing in sarcomas of uncertain origin and supports the use of immune checkpoint inhibitors as a rational therapeutic strategy for dMMR-associated sarcomas. Therefore, increased awareness of this rare association may facilitate early recognition of LS, inform personalized treatment decisions, and ultimately improve the clinical management of patients with uncommon but biologically significant malignancies.

## 8 Conclusion

This case describes a rare retroperitoneal undifferentiated pleomorphic sarcoma occurring in a patient with LS carrying a germline MLH1 mutation. The concordant dMMR/MSI-H molecular profile observed across multiple primary tumors supports the potential role of UPS as an extracolonic manifestation of LS. This report highlights the importance of considering LS in patients with sarcomas and a personal or family history of colorectal cancer and highlights the value of routine MMR/MSI testing in atypical tumors. In addition, the favorable clinical course observed with adjuvant pembrolizumab suggests that immune checkpoint inhibition may represent a promising therapeutic strategy for selected dMMR/MSI-H sarcomas. 

## Data Availability

The original contributions presented in the study are included in the article/supplementary material. Further inquiries can be directed to the corresponding author.
